# Epidemiological Trends of Human Monkeypox Cases in Northern, Southern, Western, and Eastern Regions in Europe: A Cross-Sectional Study

**DOI:** 10.1155/2022/4042962

**Published:** 2022-09-07

**Authors:** Sultan Ayoub Meo, Abdulaziz Hassan Alsomali, Abdullah Abdulrahman Almushawah, Anusha Sultan Meo

**Affiliations:** ^1^Department of Physiology, College of Medicine, King Saud University, Riyadh 11461, Saudi Arabia; ^2^College of Medicine, King Saud University, Riyadh 11461, Saudi Arabia

## Abstract

**Background:**

The growing amount of monkeypox cases in non-endemic regions raises concerns in societies as a potential pandemic. This study highlights the prevalence and epidemiological trends of a human monkeypox outbreak in various regions in Europe.

**Methods:**

This cross-sectional study was conducted in the Department of Physiology, College of Medicine, King Saud University, Riyadh, Saudi Arabia. The data about the monkeypox outbreak in European countries were recorded by the World Health Organization (WHO), and Centers for Disease Control and Prevention (CDC) reports. The period prevalence-based data were recorded from Jan 1, 2022, to July 7, 2022, and analyzed, and epidemiological trends were established in different European regions and countries.

**Results:**

In Europe, the human monkeypox rapidly spread in all the four subregions and involved 30 European countries, infecting 6077 people. The rising number of monkeypox cases is identified in Western Europe, 2599 (42.76%); Southern Europe, 1932 (31.79%); Northern Europe, 1487 (24.46%); and Eastern Europe, 59 (0.97%). In Western Europe, significant cases are found in Germany (1304), France (604), the Netherlands (352), Belgium (168), and Switzerland (131). In Northern Europe, it is found in the United Kingdom (1351), Ireland (44), Sweden (28), and Denmark (26); in Southern Europe, it is found in Spain (1256), Portugal (415), and Italy (233). However, a smaller number of cases are found in Eastern European states, Hungary (22), Poland (13), and Romania (12). The results further revealed that the number of monkeypox cases per million people in Northern Europe was 14.40%; Southern Europe, 13.49%; Western Europe, 13.26%; and Eastern Europe, 0.70%. The highest monkeypox cases per million population are found in Portugal, 40.70; Gibraltar, 29.68; Spain, 26.86; the United Kingdom, 19.90; Malta, 18.12; the Netherlands, 20.54; Germany, 15.56; Switzerland, 15.14; Belgium, 14.50; France, 9.27; and Ireland, 8.90.

**Conclusions:**

In a short period, the monkeypox cases swiftly spread in 30 non-endemic European countries and involved all four European regions. The healthcare authorities must take timely decisions to control the outbreak of human monkeypox disease, as the world cannot afford the global burden of human monkeypox outbreak as another potential pandemic.

## 1. Introduction

Monkeypox (MPX) is an emerging zoonotic disease, caused by the monkeypox virus (MPXV). The swift spread of the disease has caused an alarming situation globally [1]. The MPXV is an enveloped double-stranded DNA virus, genus Orthopoxvirus, subfamily Chordopoxvirinae, and family poxviridae [2]. Monkeypox virus belongs to the same family as smallpox, although it has a milder disease impact. The monkeypox virus is brick-shaped, with a moderately large size of about 200-250 nm, encircled by a lipoprotein [2–5].

In 1958, monkeypox was first identified as a pox-like disease in monkeys, and the disease acquired the term monkeypox. The first case of human monkeypox was reported in 1970 in the Democratic Republic of the Congo [2]. Afterward, the virus has been identified in other African countries [2, 3]. Since May 2022, monkeypox cases have been identified in many non-endemic states in European countries, the United States, Australia, Asia, and the Middle East [6].

The monkeypox disease transmits from animal to human, through direct contact with the bodily fluids, blood, or cutaneous lesions of infected animals [2]. The human-to-human transmission can result from close contact with respiratory secretions, skin lesions of an infected person, or contaminated stuff. The transmission of disease can occur via the placenta, mother to fetus, and close contact during and after birth [2]. The spread of the disease also occurs through the sexual routes [7], mainly among men who have sex with men [8, 9]; in some cases, the seminal fluid samples were positive for monkeypox viral DNA [10].

The incubation period of monkeypox is 6 to 13 days, ranging from 5 to 21 days [2]. The clinical symptoms of monkeypox disease are fever, headache, body ache, swollen lymph nodes, chills, fatigue, sore throat, nasal congestion, cough [2], and diarrhea [9]. Moreover, skin rashes, pimples, or blisters appear on the face, mouth, chest, hands, feet, and other parts of the body. The disease lasts for about 2-4 weeks. The case mortality ratio of monkeypox is 0 to 11% in the general population and higher among young children. In recent times, the mortality rate due to monkeypox disease is approximately 3-6% [2].

Since May 2022, an increasing number of human monkeypox cases are found in different countries in Europe [6]. Europe has four major topographical regions: Western Europe, Eastern Europe, Northern Europe, and Southern Europe [11]. The sudden spread of the monkeypox disease in European regions has developed a threatening and challenging situation [1]. This study aimed to highlight the prevalence of human monkeypox outbreaks, and better understand the spread of diseases in different European countries.

## 2. Materials and Methods

This cross-sectional study was performed in the Department of Physiology, College of Medicine, King Saud University, Riyadh, Saudi Arabia. This study explored the epidemiological trends of human monkeypox viral disease in various countries in different European regions.

### 2.1. Data Collections

After the study concept, one investigator was assigned to review the international websites and literature on monkeypox cases. For quality assurance, another team member was assigned to recheck the entire data for any error. Initially, two international health organizations, the World Health Organization (WHO) [12] and Centers for Disease Control and Prevention (CDC) [6], and 6 documents from PubMed [13] and Web of Science [14] were selected. However, after reviewing the detailed reports and articles, the required information about the period prevalence-based data was gathered from the World Health Organization [12] and the Centers for Disease Control and Prevention [6].

The European subregions and the population of various countries were recorded from the Worldometer [11]. The documents in PubMed [13] and Web of Science [14] were based mainly on brief reports and editorials; hence, their data and findings were not included in the analysis. The relevant literature was explored through keyword searches, including monkeypox, epidemiology, incidence, prevalence, Europe, and European countries. After the literature had been shortlisted, the appropriate period prevalence-based data were recorded and analyzed, and interpreted.

### 2.2. Ethical Statement and Statistical Analysis

The information on the monkeypox outbreak was recorded from publicly available data; hence, ethical approval was not required. In this study, the data were documented and analyzed, and the findings were expressed in numbers and percentages. The number of cases per million population was calculated by using ratios, dividing the total cases by the total population, and then multiplying it by 1,000,000. The normality of the data was assessed using the Shapiro–Wilk test. As data were not normally distributed, the Kruskal–Wallis test was used to compare the average number of cases per million among the four regions of Europe. As the Kruskal–Wallis test was significant, so pairwise comparison was performed by alpha family correction using the Bonferroni method. A *p* value ≤0.05 is considered significant.

## 3. Results

**Figure 1:**
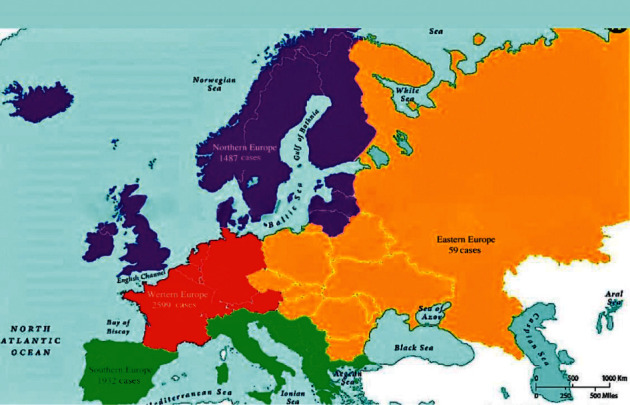
Total number of human monkeypox cases in eastern, western, southern, and northern subregions of Europe (data presented from May 7, 2022, to July 7, 2022).

**Figure 2:**
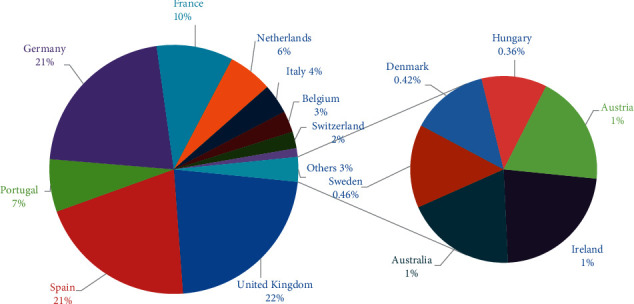
Percentage of total cases of human monkeypox disease in European countries (data presented from May 7, 2022, to July 7, 2022). The countries with less than 20 cases and their percentages are presented under other countries.

**Figure 3:**
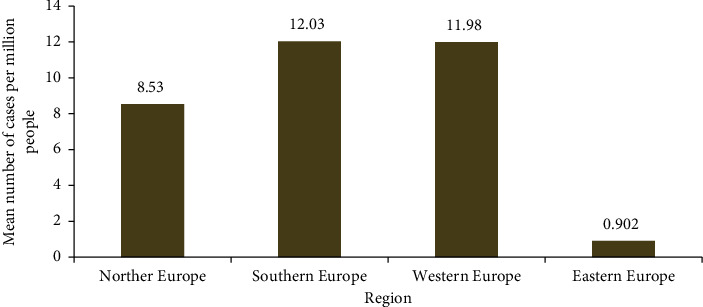
Monkeypox means cases per one million people in the different regions of Europe (cases presented from May 7, 2022, to July 7, 2022).

The data were recorded from Jan 1, 2022, to July 7, 2022. In Europe, the human monkeypox rapidly spread in all the four subregions, and involved 30 European countries, infecting 6077 people. The rising number of monkeypox cases is reported in Western Europe, 2599 (42.76%); Southern Europe, 1932 (31.79%); Northern Europe, 1487 (24.46%); and Eastern Europe, 59 (0.97%) (Table 1, Figures 1–3).

In Western Europe, the significant cases are found in Germany (1304), France (604), the Netherlands (352), Belgium (168), Switzerland (131), and Austria (31). In Northern Europe, these are found in the United Kingdom (1351), Ireland (44), Sweden (28), Denmark (26), and Norway (19). In Southern Europe, these are found in Spain (1256), Portugal (415), and Italy (233). However, a smaller number of cases are found in Eastern European states, Hungary (22), Poland (13), and Romania (12) (Table 1, Figure 2).

The highest monkeypox cases are found in the United Kingdom (1356), Germany (1304), Spain (1256), France (604), Portugal (415), Netherlands (352), Italy (233), Belgium (168), Switzerland (131), Ireland (44), Sweden (28), Denmark (26), Austria (37), Hungary (22), and in other European countries (Table 1, Figures 1 and 2). These countries have become the most affected countries worldwide. In a short period of about two months, the monkeypox cases swiftly spread in 30 non-endemic European countries and involved all four European regions.

The results revealed the point prevalence of monkeypox cases per million population in 30 countries from four subregions of Europe. The point prevalence of monkeypox cases per million people in Northern Europe is 14.40; Southern Europe, 13.49%; Western Europe, 13.26%; and Eastern Europe, 0.70% (Tables 1 and 2; Figure 1). However, the overall number of monkeypox cases in all these 30 countries in four different regions of European countries was 11.53. The results revealed that the highest monkeypox cases per million population are found in Portugal, 40.70; Gibraltar, 29.68; Spain, 26.86; the United Kingdom, 19.90; Malta, 18.12; the Netherlands, 20.54; Germany, 15.56; Switzerland, 15.14; Belgium, 14.50; France, 9.27; and Ireland, 8.90 (Table 1).  Table 3 shows the sample average rank with standard test statistics and significance level between the different regions in the European countries.

## 4. Discussion

Still, the COVID-19 pandemic continues, and the world is facing another public health threat of a global outbreak of monkeypox disease [15]. The number of monkeypox cases in non-endemic regions raises concerns in societies as a potential pandemic [1]. The monkeypox outbreak in non-endemic regions mainly in the European countries has received high attention around the world [16]. This study highlights the monkeypox outbreak and its epidemiological trends in European countries. It was identified that in Europe, human monkeypox rapidly spread in all the four subregions and involved 30 European counties, infecting 6077 people. The maximum number of monkeypox cases is identified in Western Europe, 2599 (42.76%); Southern Europe, 1932 (31.79%); and Northern Europe, 1487 (24.46%); however, the minimum number of cases is in Eastern Europe, 59 (0.97%). The significant cases are found in the United Kingdom, Germany, Spain, France, Portugal, Netherland, Italy, Belgium, and Switzerland. In a short period, the monkeypox cases swiftly spread in 30 non-endemic European countries and involved all four European regions.

Since 1970, the human monkeypox virus has been causing regular outbreaks in Central and West African nations [5]. Presently, the geographical dispersal pattern is bigger than the previous outbreaks which were more localized and occurred in under-resourced African societies [5].

The topographical dispersal of human monkeypox virus infection has been speedily shifting from endemic regions to non-endemic regions. MPXV can spread from animal to person or person to person, once an individual has close contact with the virus from an infected animal, person, or virus-contaminated materials such as clothing or linens [16]. Presently, in 2022, the human monkeypox virus swiftly spread in non-endemic regions and has knocked on the doors of developed nations in Europe, the USA, Australia, Asia, and the Middle East [6, 12]. The current volume of the outbreak is rapidly growing day-to-day, as the ecological spread continues across the world mainly the Europe [1].

Today, the world is witnessing that monkeypox cases are rapidly increasing both in endemic and non-endemic regions [17]. The World Health Organization (WHO) African regional reported that Africa has about 1821 cases in 13 countries [18]. However, in non-endemic regions of Europe, these cases are over 6000, mainly during the period from early May 2022 to July 7, 2022. The geographic spread of monkeypox to non-endemic countries in Europe is an alarming sign as in these nations, no case has ever been identified before. There are chances of local community spread of the virus in the European countries; hence, the number of cases is rapidly increasing in various states in Europe.

Over the past 5 decades, monkeypox outbreaks have occurred in various African countries, but this is the first time the disease has swiftly crossed the various continental borders and infected a large population worldwide mainly in the European countries. This is a time to learn why the disease was ignored once its cases were regularly detected in African nations. The virus has been ignored in Africa for several decades, and now, the disease is approaching the level of a potential pandemic in Europe and other parts of the globe.

### 4.1. Study Strengths and Limitations

This study highlights the epidemiological trends of the prevalence of human monkeypox virus disease in all the European regions and countries. The epidemiological data are based on the period from Jan 1, 2022, to July 7, 2022. This study attempts to harmonize the information across the regions and countries and provide a piece of additional information to highlight the epidemiological trends of the prevalence of the monkeypox outbreak in non-endemic European regions. The limitation of this study is that PubMed- and Web of Science-based literatures consist of mainly brief communication and editorials, and are hence unable to provide more detailed analyses and conclusions.

## 5. Conclusions

The human monkeypox cases rapidly spread in all the four European subregions, involving 30 European counties, infecting 6077 people from early May 2022 to July 7, 2022. A higher number of monkeypox cases is identified in Western Europe, Southern Europe, and Northern Europe; however, minimum cases are identified from Eastern Europe. A significant number of cases are found in the United Kingdom, Germany, Spain, France, Portugal, the Netherlands, Italy, Belgium, and Switzerland. The results further revealed that the number of monkeypox cases per million people was identified in Northern Europe, Southern Europe, Western Europe, and the minimum cases per million population were found in Eastern Europe. The highest number of monkeypox cases per million population is observed in Portugal, Gibraltar, Spain, the United Kingdom, Malta, the Netherlands, Germany, Switzerland, Belgium, France, and Ireland. In a short period, the monkeypox cases swiftly spread in non-endemic European countries and involved all four European regions. The healthcare authorities must take timely decisions to control the outbreak of human monkeypox disease, as the world cannot afford the global burden of human monkeypox outbreak as another potential pandemic.

## Figures and Tables

**Table 1:** European countries with population, monkeypox cases, and cases per one million people.

Subregion and country	Population	Total cases from Jan 1 to July 7, 2022	Number of cases per million people
Northern Europe			
United Kingdom	67,886,011	1351	19.90
Ireland	4,937,786	44	8.91
Sweden	10,099,265	28	2.77
Denmark	5,792,202	26	4.49
Norway	5,421,241	19	3.50
Finland	5,540,720	10	1.80
Iceland	341,243	4	11.72
Latvia	1,886,198	2	1.06
Estonia	1,326,535	2	1.51
Gibraltar	33,691	1	29.68
Subtotal	103,231,201	**1487**	**14.40**
Average	**10,326,489**	**149**	**8.53**

Southern Europe			
Spain	46,754,778	1256	26.86
Portugal	10,196,709	415	40.70
Italy	60,461,826	233	3.85
Slovenia	2,078,938	12	5.77
Malta	441,543	8	18.12
Greece	10,423,054	6	0.58
Serbia	8,737,371	1	0.11
Croatia	4,105,267	1	0.24
Subtotal	**143,199,486**	**1932**	**13.49**
Average	**17, 899,936**	**242**	**12.03**

Western Europe			
Germany	83,783,942	1304	15.56
France	65,273,511	604	9.25
The Netherlands	17,134,872	352	20.54
Belgium	11,589,623	168	14.50
Switzerland	8,654,622	131	15.14
Austria	9,006,398	37	4.11
Luxembourg	625,978	3	4.79
Subtotal	**196,068,946**	**2,599**	**13.26**
Average	**28,009,849**	**371**	**11.98**

Eastern Europe			
Hungary	9,660,351	22	2.28
Poland	37,846,611	13	0.34
Romania	19,237,691	12	0.62
Czechia	10,708,981	9	0.84
Bulgaria	6,948,445	3	0.43
Subtotal	**84,402,079**	**59**	**0.70**
Average	16,880,416	12	0.902
Grand total	**526,901,712**	**6,077**	**11.53**
Average grand total	**131,725,428**	**1,519**	**10.46**

Note: cases were searched from Jan 1 to July 7, 2022; in Europe, the first case was identified on May 7, 2022; hence the cases are presented from May 7 to July 7, 2022 (see Ref [6, 12]).

**Table 2:** European regions with the number of countries and mean monkeypox cases per one million people.

European regions	Number of countries	Mean	Standard deviation	Standard error	95% confidence interval mean		Minimum	Maximum
					Upper bound	Lower bound		
Northern Europe	10	8.53	9.4990	3.003	1.7388	15.329	1.06	29.68
Southern Europe	8	12.03	15.1052	5.3406	−.5999	24.657	0.11	40.70
Western Europe	7	11.98	6.10344	2.3068	6.3395	17.629	4.11	20.54
Eastern Europe	5	0.902	0.79386	0.3550	−0.0837	1.887	0.34	2.28
Total	30	8.9990	10.3410	1.8880	5.1376	12.860	0.11	40.70

**Table 3:** Sample average rank of the European regions.

Sample 1-sample 2	Test statistic	Standard error	Standard test statistic	Significance level	Adjusted level of significance
Eastern Europe-Southern Europe	9.175	5.019	1.828	0.068	0.405
Eastern Europe-Northern Europe	10.100	4.822	2.095	0.036	0.217
Eastern Europe-Western Europe	14.943	5.155	2.899	0.004	0.022
Southern Europe-Northern Europe	0.925	4.176	0.222	0.285	1.000
Southern Europe-Western Europe	−5.768	4.556	−1.266	0.206	1.000
Northern Europe-Western Europe	−4.843	4.338	−1.116	0.264	1.000

## Data Availability

The data may be provided on reasonable request to the corresponding author.
